# Isotopic evidence for the spatial heterogeneity of the planktonic food webs in the transition zone between river and lake ecosystems

**DOI:** 10.7717/peerj.222

**Published:** 2013-12-12

**Authors:** Hideyuki Doi, Elena I. Zuykova, Shuichi Shikano, Eisuke Kikuchi, Hiroshi Ota, Natalia I. Yurlova, Elena Yadrenkina

**Affiliations:** 1 Graduate School of Life Sciences, Tohoku University, Katahira, Aoba-ku, Sendai, Japan; 2 Institute for Sustainable Sciences and Development, Hiroshima University, Kagamiyama, Higashi-Hiroshima, Japan; 3 Institute of Animal Systematics and Ecology, Siberian Branch of Russian Academy Sciences, Novosibirsk, Russia; 4 Center for Northeast Asian Studies, Tohoku University, Kawauchi, Aoba-ku, Sendai, Japan; 5 Environmental Education Center, Miyagi University of Education, Aramaki-Aoba, Aoba-ku, Sendai, Japan; 6 Center for the Advancement of Higher Education, Tohoku University, Kawauchi, Aoba-ku, Sendai, Japan

**Keywords:** Food web, Plankton, Lake, Isotope, Spatial scale, Zooplankton, Phytoplankton, Carbon, Heterogeneity

## Abstract

Resources and organisms in food webs are distributed patchily. The spatial structure of food webs is important and critical to understanding their overall structure. However, there is little available information about the small-scale spatial structure of food webs. We investigated the spatial structure of food webs in a lake ecosystem at the littoral transition zone between an inflowing river and a lake. We measured the carbon isotope ratios of zooplankton and particulate organic matter (POM; predominantly phytoplankton) in the littoral zone of a saline lake. Parallel changes in the δ ^13^C values of zooplankton and their respective POMs indicated that there is spatial heterogeneity of the food web in this study area. Lake ecosystems are usually classified at the landscape level as either pelagic or littoral habitats. However, we showed small-scale spatial heterogeneity among planktonic food webs along an environmental gradient. Stable isotope data is useful for detecting spatial heterogeneity of habitats, populations, communities, and ecosystems.

## Introduction

Although the spatial scales of ecological processes have been recognized as important in ecology, they have presented an enormous challenge for ecologists (Levin, 1992). In many ecosystems, the organisms that comprise food webs inhabit areas that are spatially heterogeneous in terms of productivity, resource abundance, and consumer demography (Paine, 1966; Fahrig, 2001; Zambrano, Valiente & Vander Zanden, 2010). Thus, a current focus of ecological studies is the interaction among spatially heterogeneous habitats (e.g., Polis, Anderson & Holt, 1997; Thompson et al., 2001; Carlier et al., 2009). Food webs are comprised of trophic interactions from producers to top predators (Pimm, 1982) and are spatially heterogeneous and coupled with each other (e.g., Polis, Anderson & Holt, 1997; Schindler & Scheuerell, 2002).

Resources and organisms in food webs may be distributed patchily, and there is some evidence for spatial heterogeneity of food webs. Stable isotope analyses of habitat heterogeneity in lakes or lagoons (Harvey & Kitchell, 2000; Carlier et al., 2009; Zambrano, Valiente & Vander Zanden, 2010) and vertical food web data from an ocean (Richardson et al., 1998) have suggested that there may be spatial heterogeneity of aquatic food webs in these communities. Despite the increasing number of studies in the field, the small-scale spatial structure patterns of the food webs have remained unclear.

Lake ecosystems are typically classified into pelagic, littoral, and benthic habitats (Wetzel, 2001; Schindler & Scheuerell, 2002). In lake ecosystems, stable isotope analyses have demonstrated that the food-web structure is divided horizontally and vertically into several zones, such as pelagic, littoral, profundal, and benthic zones. Little evidence, however, exists for the spatial heterogeneity of lake food webs within classical lake habitats, such as littoral and pelagic zones.

In large, shallow lakes, pelagic food webs may vary spatially because of limited water movement and geomorphological complexity. In such cases, variations in environmental factors, such as water quality and temperature, are good indicators of differences between water masses or food webs. Generally, the littoral zone of a lake represents a transition between a riverine zone and a lake zone, with a longitudinal gradient of environmental factors, such as current velocity, turbidity, and photosynthetic productivity (Wetzel, 2001). In our previous studies in Lake Chany, environmental factors, such as the pH of the lake water, varied horizontally from the inflowing river to the dead end of the lake (Doi et al., 2004; Doi et al., 2006b).

**Figure 1:**
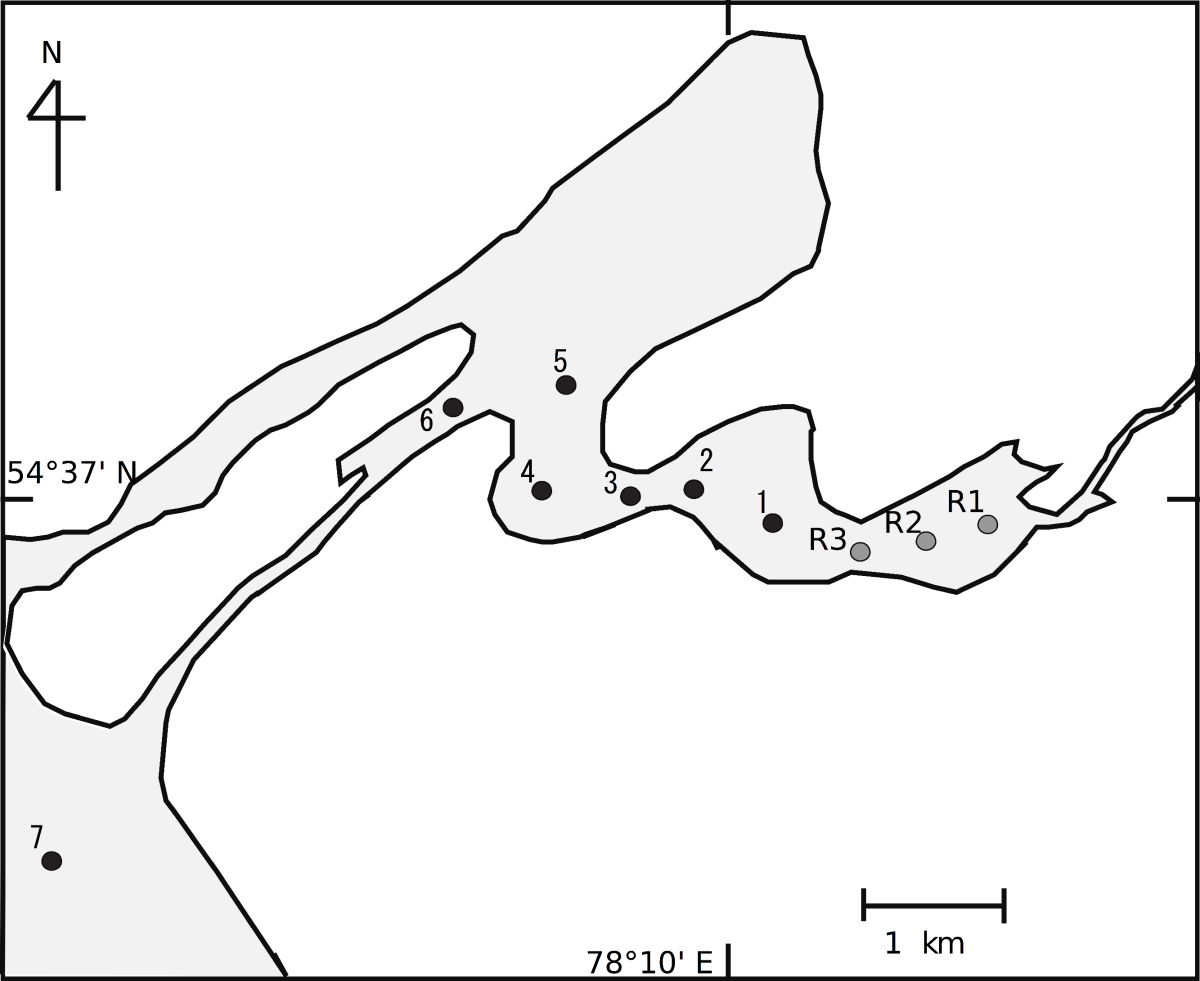
Sampling stations in the littoral transition zone between an inflowing river, the Kargat River, and Lake Chany in western Siberia.

Here, we assumed that we could use the relationship between the isotope values of consumers and environmental factors to reveal the spatial structure of planktonic food webs at a small spatial scale. Although we expect that the spatial heterogeneity of food webs is represented by changes in stable isotope composition, the association between POM and herbivores is stronger than that between POM and carnivores. This is especially true for zooplankton species in planktonic food webs, as they do not have high mobility and cannot move to connect food webs between sites. Thus, in this study, we estimated the food web in the littoral transition zone between a riverine zone and a lake zone of Lake Chany (Fig. 1).

Carbon stable isotope ratio is an excellent index for identifying the food sources of zooplankton, as minor changes in carbon isotope ratios occur with each trophic transfer (McCutchan et al., 2003). Therefore, we measured the carbon isotope ratios of carnivorous and herbivorous zooplankton species and of primary producers (particulate organic matter: POM, predominantly phytoplankton) in different parts of the littoral transition zone of Lake Chany.

**Figure 2:**
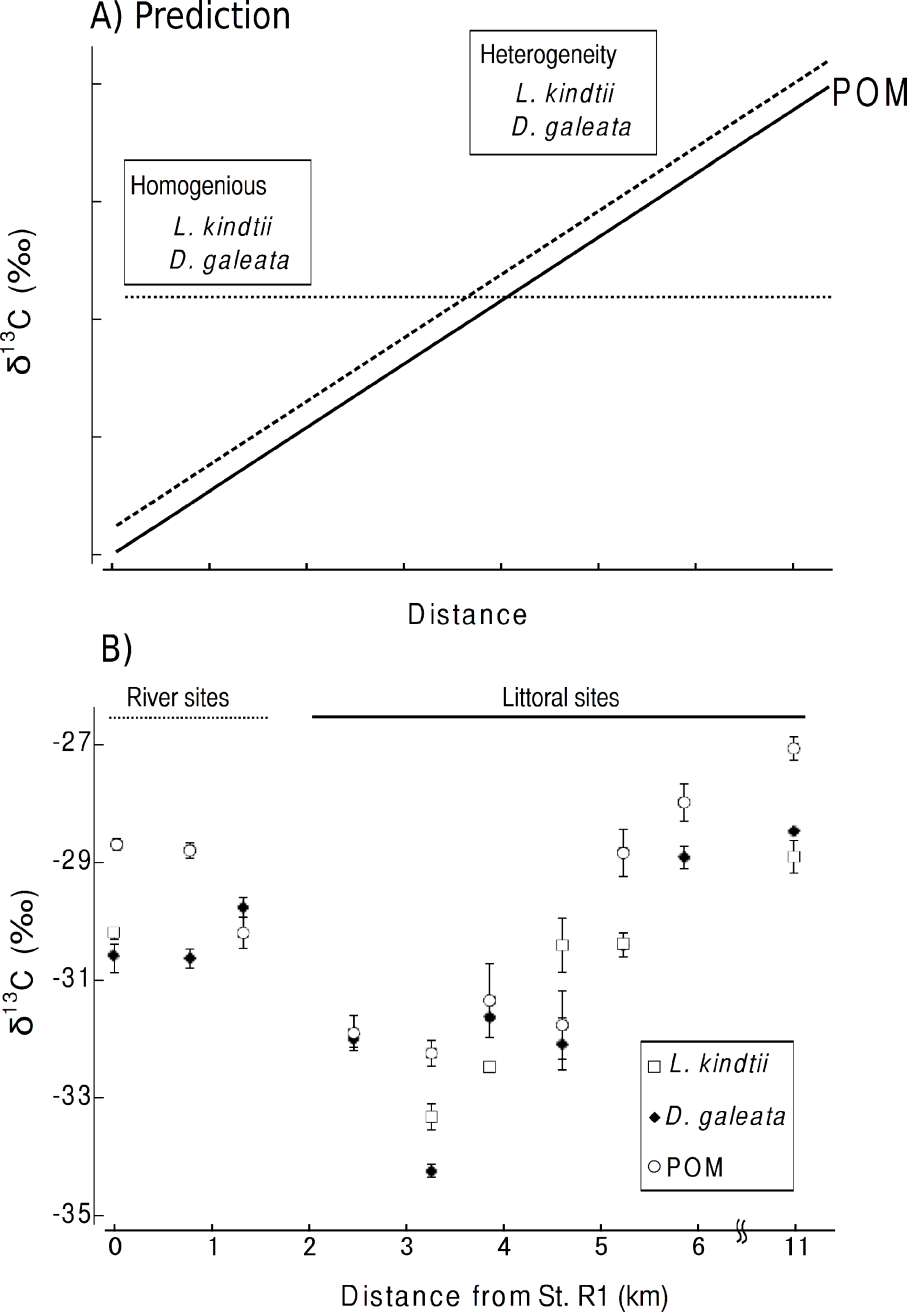
(A) Prediction of the spatial heterogeneity of food webs. The solid line indicates the trend of the carbon isotope values of particulate organic matter (POM) with distance. The broken and dotted lines represent the predicted ^13^C values for spatial heterogeneity and homogeneity of food webs, respectively. (B) Observed carbon isotope changes in POM and zooplankton species with distance from Station R1. Symbols represent mean values. Error bars are ±1 SE (*n* = 4).

Here, we considered the two following predictions. (1) The isotope ratios of herbivore and carnivore zooplankton will spatially shift parallel to those of POM within a pelagic habitat when spatial heterogeneity is present in the planktonic food web (Fig. 2A). (2) No such parallel change will occur in the herbivorous or carnivorous zooplankton or in the primary producers if the food webs were spatially homogeneous within a given area (Fig. 2A). From the isotopic evidence of the tri-trophic planktonic food webs, we can assess the spatial structure of the planktonic food webs at a small spatial scale.

## Materials and Methods

### Study area

Lake Chany is in the Novosibirsk region of the Barabinskaya lowland of western Siberia (54°30′–55°09′ N, 76°48′–78°12′ E) at an altitude of 106 m above sea level. The lake is a shallow inland saline system (average depth, 2.2 m; maximum depth, 8.5 m) that is characteristic of the western Siberian forest-steppe (Aladin & Plotnikov, 1993). Two main rivers, the Kargat and the Chulym, flow into Lake Chany. For this study, we selected study sites between the riverine and lake zones from the riverine part of the Kargat River (St. R1-3) to the mouth of Lake Chany (St. 7; Fig. 1). Sts. R1-3 were in a lotic area of the lake with detectable current velocity (approximately 20–40 cm s^−1^), while Sts. 1–7 were in a lentic area with no detectable current velocity.

### Sampling and sample preparations

All sampling was conducted between 17:00 and 21:00 h in August 2004. POM samples were obtained by filtering 100 mL surface lake water through Whatman GF/F glass fiber filters (precombusted at 500°C for 2 h). The sample water was prefiltered through plankton-net mesh (mesh size 250 µm). The samples were then treated with 1 mol L^−1^ HCl to remove bicarbonate prior to isotope measurements. Crustacean zooplankton for isotopic analysis were collected by vertically hauling a plankton net (mesh size 250 µm) from the bottom to the surface of the lake. Zooplankton was sampled four times at each site. Species were sorted manually under a stereomicroscope and washed with distilled water. We collected two crustacean zooplankton species, *Daphnia galeata* Sars, a herbivore (previously identified as *D. longispina*; Doi et al., 2006b; Zuykova et al., 2006; Zuykova, Bochkarev & Katokhin, 2013), and *Leptodora kindtii* (Focke), a carnivore, as these were the most abundant and common species at the sites (Zuykova et al., 2006). All samples were dried in a 60°C oven for 48 h and then kept frozen until isotope analyses were conducted.

The pH and electrical conductivity (EC) of the surface water at the study sites were measured using Twin pH and Twin Conductivity portable meters, (Horiba Co., Tokyo, Japan), respectively. Chlorophyll *a* concentration of the surface water was measured in five replicates at each site using a fluorescence chlorophyll *a* meter (Aqua-flow, Turner Designs, California, USA).

The carbon isotope ratios of POM and zooplankton species were measured with a mass spectrometer (DELTA plus, Finnigan Mat) connected to an elemental analyzer (NA-2500, CE Instruments, Italy). We measured 3–4 isotope samples for each site. The results are reported in delta notation: δ ^13^C = (^13^C/^12^C_sample_/^13^C/^12^C_standard_−1)⋅1000 (‰). Pee Dee Belemnite (PDB) was used as a global standard for δ ^13^C. The analysis error was ± 0.2‰ for δ ^13^C.

### Prediction and statistical analyses

Carbon stable isotope ratio is an excellent index for identifying the food sources of zooplankton, as only minor changes in carbon isotope ratios occur with each trophic transfer (McCutchan et al., 2003). Therefore, we considered the two following predictions. (1) If spatial heterogeneity was present in the planktonic food web, the isotope ratios of herbivore and carnivore zooplankton spatially would shift parallel to those of POM within a pelagic habitat (Fig. 2A). (2) If the food webs were spatially homogeneous in an area, no such parallel changes would occur in the herbivorous or carnivorous zooplankton or in the primary producers (Fig. 2A).

To test our predictions, we used a general linear model (GLM) to determine the gradient and intercept of the carbon isotope changes in the plankton. We used a one-way ANOVA and the Tukey multiple-comparison test to compare carbon isotope values among the study sites (α = 0.05). Significant ANOVA results (*p* < 0.01) were verified with the Tukey test. We also used a GLM to estimate the environmental factors, including pH, electronic conductivity, chlorophyll *a* concentration, water depth, and water temperature (Table 1), for the mean δ ^13^C values of POM. The best GLMs were selected using Akaike information criteria (AIC) values with downward stepwise procedure. All statistics were performed using R ver. 2.15.2 (R Development Core Team, 2013).

**Table 1:** Distance to each station, water depth, pH, electrical conductivity (EC), and chlorophyll *a* (Chl-*a*) at ten stations in Lake Chany. Chlorophyll *a* is reported as the mean ±1 SE (*n* = 4).

Station	Distance (km)	Depth (cm)	pH	EC (mS m^−1^)	Chl-*a* (µg L^−1^)
R1	0	170	7.9	0.224	9.4 ± 2.4
R2	0.6	150	7.7	0.238	10.0 ± 2.2
R3	1.1	140	7.9	0.238	13.3 ± 2.9
1	2.1	150	8.1	0.257	23.6 ± 5.5
2	2.8	175	8.4	0.235	22.9 ± 5.1
3	3.3	100	8.6	0.231	26.1 ± 5.6
4	4.0	150	8.8	0.227	24.2 ± 4.9
5	4.5	130	9.1	0.195	25.9 ± 5.0
6	5.0	200	9.1	0.191	21.7 ± 3.6
7	10.9	200	9.1	0.130	19.6 ± 2.7

To estimate the resource contribution to each zooplankton species, we created a simple isotope mixing model using a carbon stable isotope. We assumed two primary production sources to zooplankton; phytoplankton (POM) and terrestrial matter from macrophytes (data from H Doi, EI Zuykova, S Shikano, E Kikuchi, NI Yurlova, unpublished data).

The equation used for the isotope mixing model was:

\begin{eqnarray*} \displaystyle \delta ~{\text{}}^{13}{\mathrm{C}}_{\mathrm{zooplankton}}=f(\delta ~{\text{}}^{13}{\mathrm{C}}_{\mathrm{POM}}+\Delta ~{\text{}}^{13}\mathrm{C})+(1-f)(\delta ~{\text{}}^{13}{\mathrm{C}}_{\mathrm{Terrestrial}}+\Delta ~{\text{}}^{13}\mathrm{C})&&\displaystyle \end{eqnarray*}

where, *f* is the POM contribution to the zooplankton species. Δ ^13^C was set as −0.43‰ with reference to Grey, Jones & Sleep (2001).

## Results

The mean δ ^13^C values of POM tended to increase from St. 1 (transition part of Lake Chany) to St. 7 (mouth of Lake Chany; Fig. 2B). The mean δ ^13^C values of the riverine sites, Sts. R1-3, were not remarkably changed with high current velocity. There was a significant positive relationship between the mean δ ^13^C values of POM and those of zooplankton (GLM, *R*^2^ = 0.800, *p* < 0.001; Fig. 2B). These results may indicate that the δ ^13^C values of herbivorous and carnivorous zooplankton changed in concert with changes in the δ ^13^C values of POM in the transition zone of Lake Chany.

The isotope mixing model indicated that only the bodies of *Daphnia* in R3 were formed from 10.8% terrestrial matter and 89.2% of POM. In the other species and sites, we found that the δ ^13^C values of zooplankton species were lower than those of POM. This indicates that the majority of the planktonic food webs were derived from 100% POM.

**Figure 3:**
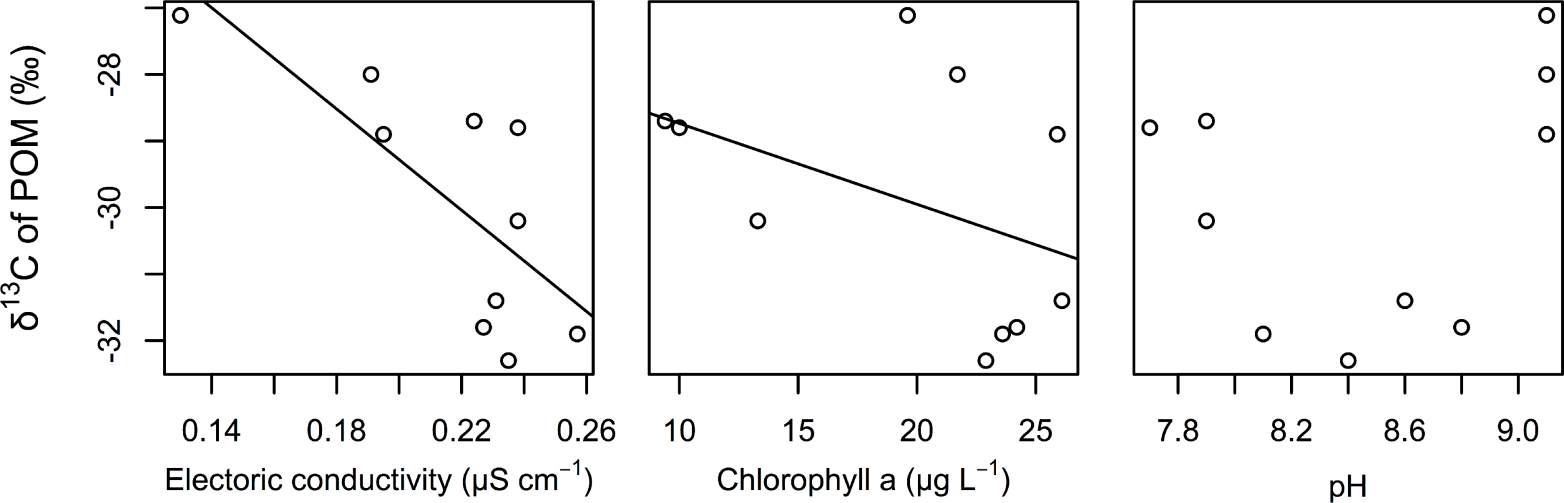
Relationships between the δ ^13^C values of POM and electrical conductivity, chlorophyll *a*, and pH of the surface water. The lines are regression lines obtained using the best GLM.

The best GLM results for the δ ^13^C values of POM indicated that electric conductivity and chlorophyll *a* were significantly related to the δ ^13^C values of POM (Table 2). Both factors were negatively correlated with the δ ^13^C values of POM (Fig. 3). We also found that the relationships between the δ ^13^C values of POM and the pH of water were not correlated.

**Table 2:** Parameters, *R*^2^ and ΔAIC values of the GLMs to explain the δ ^13^C values of POM with the environmental factors. The factors are water depth, pH, electrical conductivity (EC), and chlorophyll *a* (Chl-*a*). Bold characters mean significant parameters (*p* < 0.01).

Models	Depth	pH	EC	Chl-*a*	Intercept	*R* ^2^	ΔAIC
Full	−0.01	1.8	−23.5	−0.3	−34.3	**0.77**	0
Step 1		2	−21.6	−0.3	−37.2	**0.77**	0.36
Best			**−41.1**	**−0.2**	**−18.1**	**0.77**	0.68

## Discussion

We found parallel shifts in the δ ^13^C values of the primary producers and herbivorous and carnivorous zooplankton. Our isotope mixing model indicated that POM contributes greatly to zooplankton species at all sites. Therefore, the parallel changes in the δ ^13^C values of zooplankton and their respective POMs indicate that planktonic food webs are spatially heterogeneous in the study area (littoral sites, Sts. 1-7).

Producers and consumers that comprise food webs inhabit spatially heterogeneous habitats in many ecosystems (Paine, 1966; Polis, Anderson & Holt, 1997; Carlier et al., 2009; Zambrano, Valiente & Vander Zanden, 2010). However, food webs have mainly been studied at large scales, with little regard for the spatial organization of species (Thompson & Townsend, 2005). In these cases, the scale at which food webs are described is much larger than the scale at which the habitat of the component organisms is best described, including such features as local refugia and heterogeneous microhabitats. The spatial heterogeneity of the planktonic food webs in the present study shows that planktonic species may temporarily maintain spatial heterogeneity within microhabitats. Therefore, the spatial heterogeneity of food webs that occurs in ecosystems may be due to the utilization of spatially heterogeneous habitats by the component organisms. Food webs are composed of a number of microhabitats and are affected by the community structure of each microhabitat. Therefore, the community dynamics that affect each component microhabitat also affect the spatial structure of a food web.

Here, we only investigated zooplankton species. Therefore, conclusions regarding the whole food web in the area must be drawn with care. We found that planktonic food webs are spatially heterogeneous, due to the limited movement of zooplankton species. However, in contrast to zooplankton, fish species have higher trophic positions and can move throughout a greater area of the lake. Thus, fish species can connect the whole food web in the area, that is, they may connect the heterogeneous planktonic food-webs that we found at each site. It is important to consider the hierarchical structure of heterogeneous planktonic food-webs and that of the whole food web when evaluating the spatial structure of food webs and the connectivity between various parts of food webs. In this study, we did not evaluate the connections between heterogeneous planktonic food webs, and further study is needed in order to fully understand the spatial connectivity and structure of aquatic food webs, including mobile predators.

Habitat heterogeneity can cause similar spatial heterogeneity of food webs. The spatial structures of food webs have been considered at various spatial scales, from the local to the landscape level (Woodward & Hildrew, 2002). In this study, we found spatial heterogeneity of food webs at a small spatial scale. We expect similar food web spatial structure to occur in other ecosystems as a result of the limited movement of consumers and habitat gradients. The spatial heterogeneity of food webs should be considered at various spatial scales. Recently, stable isotopes were also used to reveal ecological niches and habitat segregations for other organisms (i.e., Miura et al., 2006). Our study is a preliminary step in demonstrating the spatial heterogeneity of food webs in various ecosystems. Stable isotope data will be useful for detecting the spatial structure of food webs in ecosystems.

In this study, we assumed that the environmental gradient caused the isotopic differences in POM in this littoral area. Although estimating the environmental factors that determine the carbon isotope value of POM was not the purpose of this study, we found that EC and chlorophyll were strongly associated with the carbon isotope value of POMs (mainly from phytoplankton). The δ ^13^C values of POM are usually correlated with δ ^13^C values of dissolved inorganic carbon (DIC) (Fry, 1996). The δ ^13^C values of DIC are generally related with conductivity in a transition zone because of changes in DIC species from CO_2_ gas to bicarbonate, which has a much higher carbon isotope value than CO_2_ gas (Bade et al., 2004; Gillikin et al., 2006). Thus, conductivity might be an important indicator of the isotope composition of POM. Spatial heterogeneity of EC is especially likely to occur in the transition zone of aquatic habitats, such as in lake-stream and river-estuary habitats. In such transition zones of aquatic habitats, the spatial structure of food webs, changes in conductivity, changes in the isotope values of POM or primary producers due to changes in the δ ^13^C values, and the concentration of DIC can be detected.

The observed negative correlation of the δ ^13^C values of POM with chlorophyll might indicate that changes in phytoplankton productivity affect the δ ^13^C values of POM. As a result of their meta-analysis, Liu et al. (2012) suggested that total phosphorous (TP), which is related to primary production by phytoplankton in lakes, is an important factor that determines the δ ^13^C values of POM in lakes. Increasing primary production would increase δ ^13^C values of phytoplankton, which is mainly composed POM, due to the limitation of carbon sources during heavy primary production (Fry, 2006). Thus, primary production would also determine the δ ^13^C values of POM in the study area. Differences in primary production have been observed in various parts of aquatic habitats, such as littoral and pelagic sites (Lauster, Hanson & Kratz, 2006) and hot spots of primary production (Payne & Moore, 2006). Such differences in primary production may also be important for the detection of the spatial structure of food webs in aquatic ecosystems.

Our study revealed the spatial structure of the planktonic food webs in varying habitats along environmental gradients, such as water chemistry and primary productivity gradients, within Lake Chany, even at a 100-m scale. Recently, other isotope species, such as sulfur and hydrogen, have been used to estimate the resources for food webs with small isotopic differences between producers and consumers (e.g., Cole et al., 2011; Doi et al., 2006a; Finlay, Doucett & McNelly, 2010). These isotopic species would also be useful for revealing spatial patterns in food webs by evaluating changes in isotope values along environmental gradients.

## Supplemental Information

10.7717/peerj.222/supp-1 Supplemental Information 1 Raw data
